# A Multifunctional Oxovanadium(V) Schiff Base Complex: Integrated Pyridoxine Sensing and Anticancer and Antimicrobial Activities

**DOI:** 10.1155/bca/8974330

**Published:** 2025-11-14

**Authors:** Tahmineh Kohanfekr, Hasan Ali Hosseini

**Affiliations:** Chemistry Department, Payame Noor University, Tehran 19395-4697, Iran

**Keywords:** anticancer, antimicrobial, electrochemical sensor, oxovanadium complex

## Abstract

The multifunctional properties of a new oxovanadium(V) complex [VO(L) (5-Cl-8-HQ)] containing a Schiff base derived from L-arginine and salicylaldehyde and 5-chloroquinolin-8-ol, were investigated in this study. The complex was characterized using elemental analysis, cyclic voltammetry, powder X-ray diffraction, as well as FTIR, UV-Vis, and ^1^H NMR spectroscopies. The electrochemical function of the complex as a sensor for pyridoxine detection showed a quasireversible behavior with an electrochemical rate constant of *k*_*e*_ = 0.133 s^−1^ in the linear range of detection of 1.0 × 10^−8^ to 1.0 × 10^−4^ mol·L^−1^, with a limit of detection of 4.24 × 10^−8^ mol·L^−1^. The complex also exhibited good activity as a potential anticancer agent in regard to the MDA-MB-231 breast cancer cell line, with IC_50_ = 35.09 ± 0.03 μg/mL with a steep hill slope of 2.575 in the dose-response curve, suggesting a sensitive cellular response to the complex, indicating promising anticancer potential. UV-Vis spectroscopy remains the method of choice for stability studies conducted under physiological conditions. The results showed a progressive complex breakdown in the cell culture medium at 37°C over 96 h, implying that its biological activity could be a mixture of the degradation products of the complex. The complex also showed antimicrobial effects at concentrations of 10 and 20 ppm against *Staphylococcus aureus*, *Enterococcus faecalis*, and *Aspergillus niger*, with the diameter of the inhibition zone (IZ) increasing with increasing complex concentration. The combination of these electrochemical sensing properties with the dual therapeutic potential as an anticancer and antimicrobial agent places this class of vanadium complexes in a versatile position for further development in both analytical and medicinal fields.

## 1. Introduction

The 21st century has witnessed an unprecedented convergence of global health challenges that demand innovative scientific solutions [1]. Among these, cancer remains a major cause of global mortality, and antimicrobial resistance is a major challenge to modern medicine. The World Health Organization has identified antimicrobial resistance, which includes resistance in bacterial, viral, and fungal pathogens, as one of the top 10 threats to global public health, signifying the importance of research on novel therapeutic alternatives to antibiotics [2]. Many researchers have begun to examine inorganic medicinal chemistry in response to these challenges. Among these, metal-based complexes have been especially promising because of the advantages of metal complexes over organic compounds in terms of their unique chemical behavior and biological activity [3]. Vanadium complexes are of particular interest because of their coordination chemistry [4], redox properties [5], and interesting biological behavior [6]. Vanadium complexes exhibit distinct geometries and are thermodynamically stable in the +4 and +5 oxidation states [7]. Notably, vanadium complexes, especially those containing azomethine (-CH=N- or >C=N-) functional groups, are very stable and have well-defined geometries compared to simple vanadium salts, addressing key concerns in drug development [8]. Vanadium complexes show biological activity, ranging from insulin-mimetic applications useful in the treatment of diabetes [9, 10] to anticancer effects via multiple mechanistic pathways of action within a cell [11, 12] and the ability to manipulate cellular enzymatic reactions that are often critical to the cellular function of the organism [13]. In addition, their electrochemical characteristics render them excellent materials for sensing applications, particularly in the analysis of pharmaceuticals and biomedical diagnostics [14]. Therefore, it is important to evaluate the stability of vanadium complexes in biological systems and aqueous solutions, as their behavior under relevant physiological conditions can influence the interpretation of their biological activities and therapeutic potential [15, 16].

On the other hand, hydroxyquinoline derivatives, a significant group of heterocyclic molecules, have garnered attention due to their diverse array of medicinal properties [17] such as antioxidant, antibacterial, antifungal [18], anticancer, anti-inflammatory, antiviral, and antiparasitic effects [19]. Their ability to coordinate with metal ions has led to their investigation into treating metal overload disorders and making them valuable scaffolds in drug discovery and development across multiple therapeutic areas [16]. The use of Schiff base ligands and hydroxyquinoline derivatives for coordination with the vanadium centers affords complexes with improved electrocatalytic properties, stabilizes vanadium in high oxidation states, and modifies the electron density around the metal center, affecting the redox potentials and reaction kinetics [20]. The reversible V(V)/V(IV) redox couple facilitates electron transfer processes that are fundamental to catalytic reactions, enabling applications ranging from drug detection to energy conversion [21].

The main purpose of this study is to develop versatile vanadium-based platforms that can serve as multipurpose tools in modern healthcare, offering integrated solutions that are more efficient and cost-effective than the use of separate compounds for each application.

## 2. Experimental Section

### 2.1. Materials and Methods

The chemicals used in this study, obtained from Merck, included methanol, vanadyl acetylacetonate, ethanol, 2-hydroxybenzaldehyde, 2-amino-5-guanidinopentanoic acid (L-arginine), 5-chloro-8-hydroxyquinoline, pyridoxine hydrochloride (vitamin B_6_), KCl, iron (II) sulfate, graphite powder, mineral oil, sodium benzoate, ascorbic acid, thiamine hydrochloride (vitamin B_1_), L-lysine hydrochloride, and histidine, which were used without any further purification. Infrared spectral data were obtained using a Thermo Nicolet AVATAR 370 FTIR spectrophotometer in the range of 400–4000 cm^−1^. A VARIAN-INOVA 300 MHz spectrometer was used to record the ^1^H NMR spectra, and all samples were analyzed using dimethyl sulfoxide (DMSO)-d_6_. Melting points were obtained using a Barnstead Electrothermal 9200 instrument. The elemental composition was determined using a Thermo Flash Elemental Analyzer 1112 EA. UV-Vis absorbance spectra were recorded in dry DMSO and cell medium culture (Dulbecco's Modified Eagle Medium [DMEM]) using a CECIL 8000 spectrophotometer. Electrochemical measurements were conducted using a Metrohm polarograph VA Computrace 797 and a Metrohm Autolab PGSTAT101 potentiostat. Powder X-ray diffraction (PXRD) analysis was performed using a GNR Explorer Theta/Theta X-ray Diffractometer (Serial no. 16015 EXP01). The measurements were carried out using Cu Kα radiation (*λ* = 1.54060 Å) at room temperature (25°C) over a 2*θ* range of 10°–45° with a step size of 0.01°. The pH values were checked using an Activon 210 ionometer that was equipped with an AEP 321 glass/Ag/AgCl electrode.

### 2.2. Synthesis of Oxido(5-Chloroquinolin-8-Olato-κ^2^N,O){2-[(2-Oxidobenzylidene-κO)Amino-κN]-5-Guanidinopentanoato-κO}Vanadium(V)

The vanadium(V) complex and Schiff base ligand (L) were prepared according to our previous report [22]. In brief, the process entailed dissolving ligand (L) (1.12 g, 4 mmol) and vanadyl acetylacetonate (1.06 g, 4 mmol) in 60 mL of methanol and refluxing for 24 h. The reaction mixture was initially blue and became red–brown upon the addition of 5-chloroquinolin-8-ol (0.71 g, 4 mmol). The solution was refluxed for a further 24 h. The solvent was halved, and the precipitate was collected via filtration. The filtrate was then placed in a refrigerator overnight, leading to the formation of a dark pseudocrystalline solid. These solids were subsequently filtered, rinsed three times with 5 mL of ethanol, and left to dry in air at room temperature. This gave 0.9 g of the complex, which amounted to a 43.2% yield.

### 2.3. 3-(4,5-Dimethylthiazol-2-yl)-2,5-Diphenyltetrazolium Bromide (MTT) Assay and Solution Stability

The cytotoxicity of the [VO(L) (5-Cl-8-HQ)] complex was evaluated using the MTT assay [23] over a 24-h period. This method required the use of DMEM from Grand Island, NY, USA; fetal bovine serum (FBS) from Gibco (lot: 2378406); penicillin–streptomycin (100X) (Bl-1203) from Bio-IDEA; MTT reagent from Sigma-Aldrich (lot: MKCK7253); DMSO and MDA-MB-231 human breast cancer cell line from the Pasteur Bank in Tehran; and an Eliza reader (Tecan 200).

For cell culture, MDA-MB-231 breast cancer cells were grown in DMEM supplemented with 10% FBS and 1% antibiotic/antimycotic solution (Invitrogen Corp). The cells were maintained at 37°C in a humidified atmosphere containing 5% CO_2_. Five different samples were tested: four direct concentrations of [VO(L) (5-Cl-8-HQ)] at 150, 100, 40, and 10 μg/mL, plus one extracted sample prepared by incubating 100 μg/mL of the complex in a cell culture medium (DMEM) at 37°C for four days following ISO 10993 guidelines [24]. Cells were seeded in 96-well plates at a density of 10 × 10^4^ cells per well and incubated at 37°C for 24 h. Subsequently, the culture medium was removed and replaced with 100 μL of the prepared sample containing 10% FBS. After 24-h treatment, cell viability was assessed using the MTT reagent (0.5 mg/mL), and the absorbance was measured at 570 nm using an ELISA reader. For comparative purposes, negative control wells (cells with the culture medium) and positive control wells (cells with 10% DMSO to induce maximum cell death) were included in the experimental setup.

Stabilities of V(V) complexes were studied by electronic absorption spectroscopy at 37°C in a cell culture medium (DMEM) that was additionally supplemented with 10% FBS and 2-[4-(2-hydroxyethyl)piperazin-1-yl]ethanesulfonic acid (HEPES) (10 mM) to maintain the pH at 7.4.

### 2.4. Antimicrobial Assessment

The antimicrobial properties of the compound [VO(L) (5-Cl-8-HQ)] were evaluated using the agar well diffusion technique [25]. This assessment targeted gram-positive bacterial strains, specifically *Staphylococcus aureus* ATCC 25923 and *Enterococcus faecalis* (Group D *Streptococcus*) ATCC 11700. The bacterial cultures were standardized to 0.5 McFarland turbidity and subsequently spread on Mueller–Hinton agar plates. Wells were created in agar and filled with 10 and 20 ppm solutions of the complex dissolved in 4%-5% DMSO. The inoculated plates were incubated at 30 ± 5°C for approximately two days. Antimicrobial efficacy was determined by measuring the diameter (mm) of the growth inhibition zones (IZs). Chloramphenicol served as a reference standard in this assay.

To assess the antifungal activity, the complex was tested against *Aspergillus niger* ATCC 16404. This evaluation also utilized the agar well diffusion method, but with Rose Bengal Agar plates. The plates were inoculated with an *Aspergillus niger* spore suspension at a concentration of 1 × 10 CFU/mL, filled with the complex solution, and incubated for five days at 30 ± 5°C to observe any antifungal effects.

### 2.5. Electrochemical Measurements

Voltammetric determinations were performed in a 30 mL temperature-controlled glass cell at 25°C. A modified carbon paste working electrode, Ag/AgCl reference electrode, and platinum wire counter electrode comprised a three-electrode setup. Measurements were conducted with −1.0 V to +1.0 V potential window at 100 mV·s^−1^ scan rate, in the absence of stirring and without deaerating the solution throughout the experiments. A 0.1-mol·L^−1^ KCl solution was used as the supporting electrolyte for all experiments. The standard pyridoxine solution, with a concentration of 0.01 mol·L^−1^, was prepared by accurately dissolving pyridoxine hydrochloride in 100 mL of a KCl solution. For the preparation of the modified carbon paste electrode (CP-[VO(L) (5-Cl-8-HQ)]), 0.2 g of the oxovanadium(V) complex was mixed with 2 g of graphite powder, and then 1.0 g of mineral oil was added. This modified paste was then packed into a plastic cylindrical tube (4 mm outer diameter and 2 mm inner diameter) with external electrical contact with the copper wire. The electrode surface was pressed against a glass plate to ensure a good packing.

Cyclic voltammetry of a 10^−3^ M solution of the complex [VO(L) (5-Cl-8-HQ)] was conducted in dry DMSO containing 0.1 M tetrabutylammonium bromide as the supporting electrolyte at 25°C. Measurements were performed using a glassy carbon working electrode, Ag/Ag^+^ reference electrode, and platinum wire counter electrode at a scan rate of 100 mV·s^−1^ over a potential range of −1.5 to +1.5 V versus Ag/Ag^+^.

## 3. Result and Discussion

A hexa-coordinated oxovanadium complex [VO(L) (5-Cl-8-HQ)] was prepared by combining a Schiff base ligand and 5-chloroquinolin-8-ol with vanadyl acetylacetonate in a methanolic medium. The resulting dark brown pseudocrystalline substance exhibited good solubility in a range of organic solvents, including CH_2_Cl_2_, CH_3_OH, C_2_H_5_OH, and DMSO, while showing moderate solubility in aqueous solutions.

**Scheme 1:**
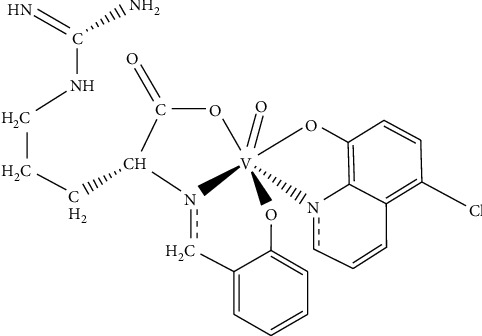
Proposed complex structure.

Elemental analysis confirmed the proposed molecular structure, indicating a 1:1 molar ratio of ligand to metal in the hexa-coordinated vanadium complex, as depicted in Scheme 1. Schematic diagrams of the synthesized complex and ligand (L) have been previously reported [22].

### 3.1. Spectral Characterization

**Figure 1:**
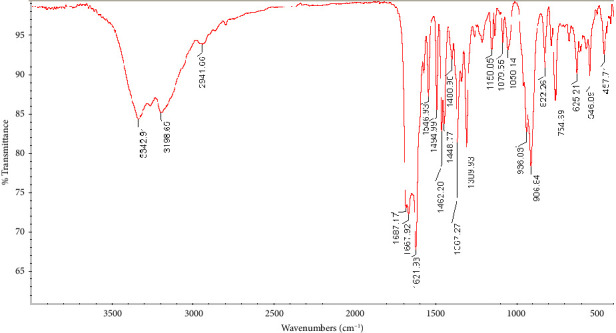
FTIR spectrum of the complex [VO(L) (5-Cl-8-HQ)].

The spectroscopic data of the ligand (L) are available in previous work [22]. The FTIR spectrum of the complex [VO(L) (5-Cl-8-HQ)] is shown in Figure 1. The spectrum revealed NH stretching from the Schiff base aliphatic chain at 3342 cm^−1^ and 3198 cm^−1^. In comparison with the ligand spectrum reported in Reference 22, the absorption bands corresponding to the OH stretches of the phenol and acid groups at 3411 and 3370 cm^−1^, respectively, were absent in the complex. In addition, the carbonyl stretching frequency observed at 1592 cm^−1^ in the ligand shifts to 1546 cm^−1^ in the complex, which is consistent with the monodentate COO-oxygen metal configuration. Similarly, the azomethine group vibration found at 1633 cm^−1^ in the ligand spectrum shifts to 1621 cm^−1^ in the complex, suggesting nitrogen coordination [26]. The CH_2_ stretch vibrations exhibit bathochromic shifts to 2941 cm^−1^, indicating ligand–metal binding. Aromatic C=C bonds appeared at 1494 cm^−1^ and 1468 cm^−1^, while a strong C-Cl stretch from quinoline was observed at 754 cm^−1^. The V=O band at 906 cm^−1^, along with V-O and V-N stretches at 625 cm^−1^ and 546 cm^−1^, confirms quinoline coordination [27]. The infrared spectroscopy results confirmed that the molecule attaches through multiple points: it uses both imine nitrogen and carboxylate oxygen atoms from its three-pronged Schiff base portion, while the quinoline segment binds through its nitrogen and oxygen atoms.

**Figure 2:**
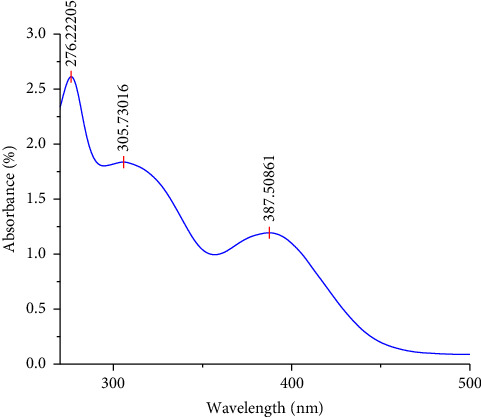
UV-Vis spectrum of the 10^−3^ M complex [VO(L) (5-Cl-8-HQ)] in dry DMSO.

The UV-Vis spectra of the 10^−3^ M [VO(L) (5-Cl-8-HQ)] complex in dry DMSO (Figure 2) show the expected *π* ⟶ *π*^∗^ (276 nm) and *n* ⟶ *π*^∗^ (305 nm) transitions due to metal–ligand bonding. The presence of a ligand-to-metal charge transfer (LMCT) transition is demonstrated by an absorption band at 387 nm. This low-energy transfer (*Pπ* ⟶ 3d) confirms that the ligand was bound to the metal [28]. According to the literature [29], the shift of the LMCT absorption band from 320 nm in VO(acac)_2_ to 387 nm in the complex provides clear evidence for the coordination of the ligands to the central vanadium atom.

**Figure 3:**
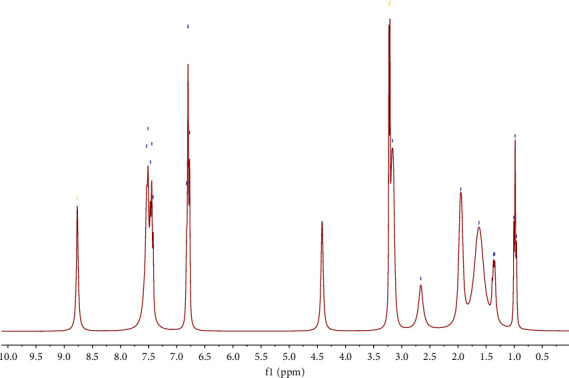
^1^H NMR spectrum of the complex [VO(L) (5-Cl-HQ)] in DMSO-d_6_.

^1^H NMR spectra were recorded in DMSO-d_6_, and the complex proton spectrum is shown in Figure 3. The NMR spectrum reveals several diagnostic signals: the chain protons of arginine resonate between *δ* = 1.37–3.20 ppm, and a singlet at *δ* = 8.77 ppm corresponding to the imine proton (CH=N) provides evidence of arginine structure. Aromatic proton peaks can be observed from *δ* = 6.78 to *δ* = 7.48 ppm [30].

According to the ^1^H NMR Schiff base ligand spectrum [22], the disappearance of the ^1^H NMR signals, corresponding to the carboxylic acid proton (14.29 ppm) and phenolic OH proton (9.45 ppm) and azomethine proton resonance (8.45 ppm) in the complex spectrum, indicates the coordination of the ligand to the metal center through the O-N-O donor atoms.

Comprehensive spectroscopic data and melting point are summarized in Table 1. Chlorine substitution at Position 5 of 8-hydroxyquinoline significantly affected its ^1^H NMR, UV-Vis, and FTIR spectra owing to its electron-donating behavior through resonance. This results in upfield shifts for aromatic ring protons, increased shielding of nearby protons in the ^1^H NMR spectra, and blue shifts in FTIR and UV-Vis spectra [31].

**Figure 4:**
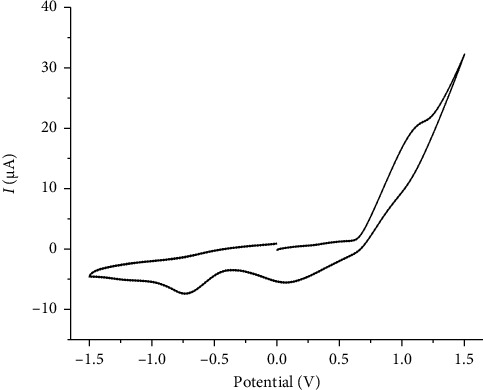
Cyclic voltammogram of the 10^−3^ M solution of the complex [VO(L) (5-Cl-8-HQ)] in dry DMSO with 0.1 M tetrabutylammonium bromide as the supporting electrolyte at scan rates of 100 mV/s.

Cyclic voltammetry (Figure 4) was performed on a 10^−3^ M solution of the complex [VO(L) (5-Cl-8-HQ)] in dry DMSO, with 0.1 M tetrabutylammonium bromide as the supporting electrolyte. The measurements were carried out using a glassy carbon working electrode, Ag/Ag^+^ reference electrode, and platinum wire counter electrode. The scan rate was 100 mV·s^−1^, and the potential range was from −1.5 to +1.5 V vs. Ag/Ag^+^. The voltammogram showed a prominent cathodic peak at approximately −0.7.3 V, which was related to the single-electron reduction of vanadium(V) to vanadium(IV) [32, 33]. The increase in anodic current at potentials higher than +1.1 V is due to the oxidation processes, which are the main cause of the coordinated-ligand framework. Previous voltammetric works of VO(acac)_2_ have led to the point of no anodic peak and a cathodic peak at a lower potential [22].

### 3.2. PXRD

**Figure 5:**
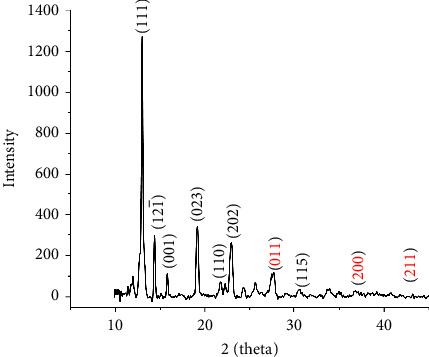
XRD pattern of [VO(L) (5-Cl-HQ)].

The PXRD pattern of the complex [VO(L) (5-Cl-HQ)] revealed well-defined crystalline peaks (Figure 5), confirming the formation of a crystalline coordination complex. The diffractogram exhibited a characteristic intense peak at 2*θ* = 12.97° (d-spacing = 6.83 Å), which served as the primary reflection with a 100% relative intensity. Additional significant peaks were observed at 2*θ* = 23.04° (d-spacing = 3.86 Å, 18.34% relative intensity), 19.21° (d-spacing = 4.62 Å, 24.76% relative intensity), and 14.37° (d-spacing = 6.16 Å, 23.45% relative intensity). The XRD pattern shows numerous peaks closely resembling those of the VO_2_ phase at 27.79°, 36.83°, and 42.20°, which are representative of the (011), (200), and (211) planes, respectively, and are well in agreement with those of the standard JCPDS card (no. 43-1051) [34, 35]. The pattern displayed multiple well-resolved reflections throughout the measured range, indicating a complex crystal structure consistent with the proposed coordination geometry.

The average crystallite size was calculated using the Debye–Scherrer equation (equation (1)) applied to the four most intense peaks at 2*θ* = 12.97°, 23.04°, 19.21°, and 14.37° (*D* is the particle size [nm], *k* = 0.94 is a crystallized form factor, *λ* is the wavelength [0.154 nm], *β* represents the highest width at half maximum of the peak [rad], and *θ* is the diffraction angle) [36].

(1) $$\begin{array}{c} D=\frac{K\lambda }{\beta \cos\theta }. \end{array}$$

The calculated crystallite sizes ranged from 15.7 to 45.1 nm, with an average crystallite size of 34.1 nm, indicating that the powder sample consisted of small crystalline domains. This crystallite size is typical for coordination complexes and reflects the microcrystalline nature of the synthesized materials [37]. The sharp and well-defined nature of the diffraction peaks confirmed the good crystallinity and structural order of the synthesized compound.

### 3.3. Electrochemical Studies of the VO(IV)/VO(V) Redox Pair

**Figure 6:**
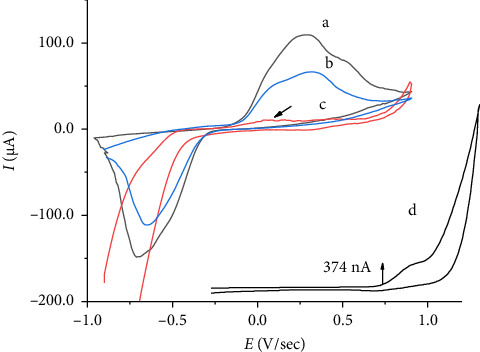
Cyclic voltammograms were recorded at a scan rate of 100 mV·s^−1^: (a) 4.0 × 10^−4^ mol·L^−1^ pyridoxine at the CP-[VO(L) (5-Cl-8-HQ)] electrode in a 0.10 mol·L^−1^ KCl solution, (b) the CP-[VO(L) (5-Cl-8-HQ)] electrode in the absence of pyridoxine, (c) an unmodified carbon paste electrode in a 0.10 mol·L^−1^ KCl solution, and (d) 4.0 × 10^−4^ mol·L^−1^ pyridoxine at an unmodified carbon paste electrode in a 0.10 mol·L^−1^ KCl solution [41].

The electrochemical properties of the modified electrode were evaluated using a scan rate of 100 mV·s^−1^ in a 0.1 mol·L^−1^ KCl solution at pH 6. It has been found that KCl and NaNO_3_ solutions are the most suitable supporting electrolytes for voltammetric determinations because they are more stable than phosphate and acetate buffers. In phosphate buffer, the voltammetric response was not stable and did not yield identifiable redox peaks. The reason for this is that vanadium cations form phosphate salt precipitates on the electrode surface, given their pKsp value of 25.1, as indicated in reference [38].  Figure 6(b) shows a cyclic voltammogram at the CP-[VO(L) (5-Cl-8-HQ)] electrode in the absence of pyridoxine with two well-defined peaks: an anodic peak at 0.329 V and a cathodic peak at −0.661 V (vs. Ag/AgCl) obtained at a scan rate of 100 mV·s^−1^ in a 0.1 mol·L^−1^ KCl solution at pH 6. The electrochemical reactions are typically attributed to a quasireversible process involving the transfer of a single electron in the reduction-oxidation of the V^V^O/V^IV^O redox pair at the complex's metallic center.  Figure 6(a) presents cyclic voltammograms characterizing the oxidation of 4.0 × 10^−4^ mol·L^−1^ pyridoxine at the CP-[VO(L) (5-Cl-8-HQ)]-modified carbon paste electrode in 0.10 mol·L^−1^ KCl solution. Pyridoxine oxidation occurs at 1.03 V (Figure 6(d)) [39]. However, the oxidation peak of pyridoxine overlaps with the strong redox process of the V^IV^O/V^V^O couple at 0.27 V. Both the anodic and cathodic peak currents of the complex significantly increased in the presence of vitamin B_6_. The presence of a shoulder in the anodic peak in Figures 6(a) and 6(b) indicates that the electrochemical behavior is more complex, involving multiple oxidation events or interactions occurring at or near the electrode surface [40]. The proposed electrocatalytic process for the CP-[VO(L) (5-Cl-8-HQ)] electrode involves two fundamental processes:

• Initial electrode surface modification, in which ligands stabilize the vanadium center and facilitate electron transfer
• Vanadium redox cycling is typified by the following reaction sequence:
  (2) $$\begin{array}{c} {\text{VO}}^{V}+e^{-}⟶{\text{VO}}^{\text{IV}-}, \end{array}$$
  (3) $$\begin{array}{c} {\text{VO}}^{\text{IV}-}+{\text{pyridoxine}}_{\text{redoxed}}⟶{\text{VO}}^{V}+{\text{pyridoxine}}_{\text{oxidized}}+H^{+}. \end{array}$$

The vanadium center undergoes a reversible redox transition during pyridoxine oxidation and acts as a redox mediator, lowering the activation energy of pyridoxine oxidation.

An investigation was performed to study the influence of scan rates ranging from 25 to 250 mV·s^−1^ on the voltammetric properties of CP-[VO(L) (5-Cl-8-HQ)] in a 1.0 × 10^−4^ mol·L^−1^ pyridoxine solution. The results indicated that increasing scan rates were associated with increasing intensities of anodic and cathodic peak currents. In addition, the scan rate increase caused a shift in the peak potentials, with anodic peaks moving toward more positive potentials and cathodic peaks moving toward less negative potentials, which is characteristic of quasireversible electrochemical behavior [40].

**Figure 7:**
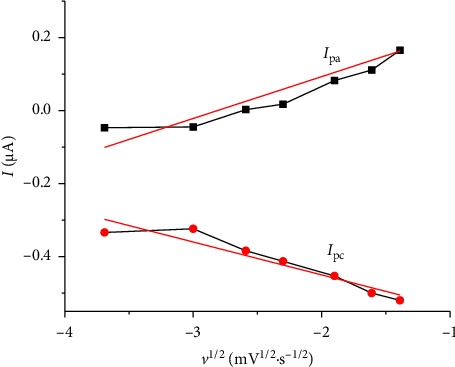
Relationship between *I*_pa_ and *I*_pc_ peak currents and the square root of scan rate for CP-[VO(L) (5-Cl-8-HQ)] between −1.0 V and 1.0 V potential range using 0.1 mol·L^−1^ KCl electrolyte.

The research revealed a linear relationship between the anodic peak current and the square root of scan rate (Figure 7), as represented by the equation *I*_pa_ (μA) = 129.84 + 1917 *υ*^1/2^ (mV^1/2^·s^−1/2^), with a correlation coefficient value of 0.966 (*n* = 7). This relationship indicated that pyridoxine oxidation proceeds via a diffusion-controlled mechanism. The kinetic parameters of the electrode were assessed using Laviron's method [42], which allows the determination of the electron transfer coefficients (*α*_*a*_ and *α*_*c*_) and the apparent electrochemical rate constant (*k*_*e*_) from the plots of Ep vs. log *v* (Figure 6). In experiments with a high scan rate, they plotted two straight lines, with the slopes being −2.303RT/*α*_*c*_nF for the cathodic process and 2.303RT/(1 − *α*_*a*_)*nF* for the anodic process. For the single-electron transfer process (*n* = 1), the calculated values were 0.223 for *α*_*a*_ and 0.285 for *α*_*c*_. The apparent rate constant, determined using *k*_*e*_ = 2.303*α*_*c*_*nFv*_*o*_/RT, was 0.133 s^−1^, confirming a quasireversible electron transfer process with moderate kinetics, tending toward irreversibility at faster scan rates.

**Figure 8:**
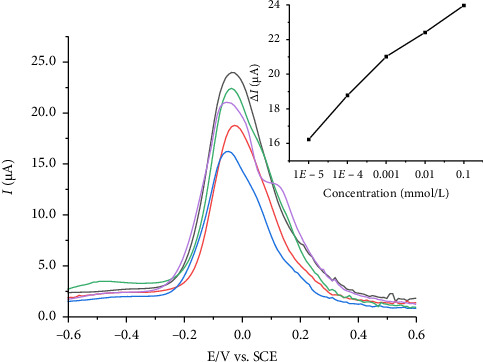
Differential pulse voltammetric at 100 mV·s^−1^ for CP-[VO(L) (5-Cl-8-HQ)] at different concentrations of pyridoxine: 1.0 × 10^−4^, 1.0 × 10^−5^, 1.0 × 10^−6^, 1.0 × 10^−7^, and 1.0 × 10^−8^ in 0.10 mol·L^−1^ KCl solution. The relationship between the pyridoxine concentration and anodic peak current is shown in detail in the derived calibration curve.

After optimizing the experimental conditions (scan rate of 100 mV·s^−1^ and pH = 6), differential pulse voltammetry was employed to investigate the electrode response to different concentrations of pyridoxine.  Figure 8 presents the linear variation of the anodic peak current with pyridoxine concentration in the range of 1.0 × 10^−8^ to 1.0 × 10^−4^ mol·L^−1^. The regression equation was *I*_pa_ (μA) = 31.95 + 1.911 (pyridoxine) (mol·L^−1^) with a correlation coefficient of 0.983 (*n* = 3), indicating reliable performance. The detection limit, calculated as three times the signal blank/slope ratio [43], was 4.24 × 10^−8^ mol·L^−1^ at pH 6 in a KCl solution. The results indicate that the oxidation of pyridoxine repeatedly occurs near 0 V vs. SCE across all concentrations studied with small shifts in potential, reflecting a stable and clearly defined electrode response. Repeatability measurements using 1.0 × 10^−3^ mol·L^−1^ pyridoxine in 0.1 mol·L^−1^ KCl solution (pH 6) provided a relative standard deviation of 3.03% for 10 repeated measurements, indicating good reproducibility for repeated measurements.

The modified electrode's selectivity for pyridoxine was evaluated by examining the interference of compounds that are normally present alongside pyridoxine in pharmaceutical products, particularly other B vitamins. This interference study was carried out with differential pulse voltammetry in 0.10 mol·L^−1^ KCl solution (pH 6.0) containing 1.0 × 10^−4^ mol·L^−1^ pyridoxine in the presence of equal concentrations (1.0 × 10^−4^ mol·L^−1^) of various possible interferents: sodium benzoate, L-lysine hydrochloride, histidine, Fe(II), ascorbic acid, and thiamine hydrochloride (vitamin B_1_).

Among the interferents, sodium benzoate, Fe(II), vitamin B_1_, and ascorbic acid caused the most negative shift potential and decreased the peak current. L-Lysine hydrochloride and histidine exerted moderate interference (6.5%), representing a moderate impact. Despite these interferences, the anodic peak response of pyridoxine at 1.0 × 10^−4^ mol·L^−1^ remained distinguishable, indicating that the sensor maintained reasonable selectivity and sensitivity under the tested conditions.

### 3.4. Stability and Viability Studies

**Figure 9:**
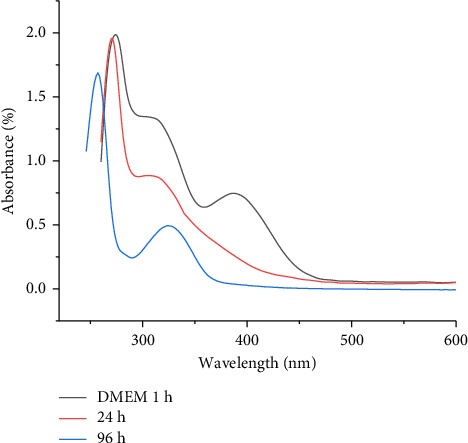
Time-dependent UV-Vis spectra of [VO(L) (5-Cl-8-HQ)] at 40 μg/mL concentration in DMEM medium supplemented with 10% FBS and 10 mM HEPES buffer, at pH 7.4, maintained at 37°C for 1 h, 24 h, and 96 h.

The stability of 40 μg/mL [VO(L) (5-Cl-8-HQ)] was assessed in DMEM medium supplemented with 10% FBS and 10 mM HEPES buffer, at pH 7.4 using UV-Vis spectroscopy, maintained at 37°C over a 96 h period. The results indicate structural changes or decomposition of the coordination complex under physiological conditions. Notably, the LMCT band at 387 nm, which was clearly visible in DMSO solution (Figure 2), was absent in DMEM after 24 h, indicating an alteration in the coordination framework upon interaction with the biological medium. Monitoring the changes over time (1 h, 24 h, and 96 h) revealed a gradual decrease in the absorption peaks at approximately 300 nm, with the intensity dropping to 1.35 at 1 h, to 0.9 at 24 h, and finally to 0.45 after 96 h (Figure 9). These changes result from alterations in the structure of the complex or its breakdown, leading to the formation of decomposition products [44].

**Figure 10:**
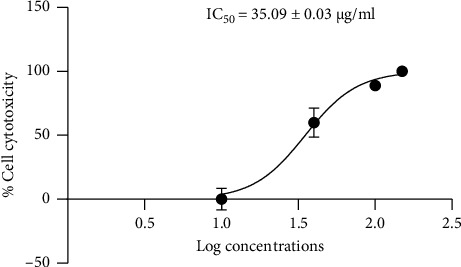
IC_50_ value of the inhibitor.

**Figure 11:**
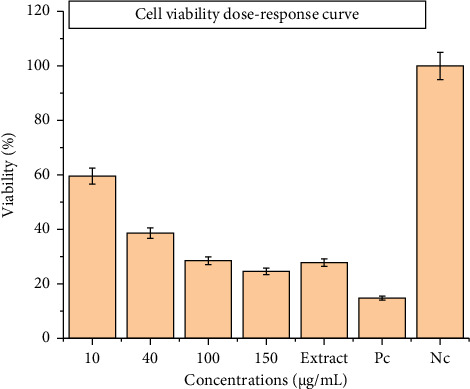
The cell viability effects of [VO(L) (5-Cl-8-HQ)] on MDA-MB-231 cell line after 24 h; Pc: positive control (cells with 10% DMSO), Nc: negative control (cells with the culture medium).

**Figure 12:**
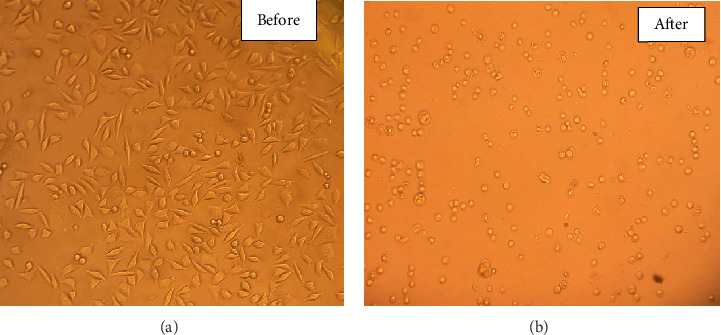
Anticancer activity of [VO(L) (5-Cl-8-HQ)] at 150 μg/mL concentration on the MDA-MB-231 cell line, before and after 24 h of treatment.

Cytotoxicity assays demonstrated significant biological effects upon exposure to [VO(L) (5-Cl-8-HQ)], although stability analyses suggest that these effects are primarily due to the decomposition products of the complex rather than the intact molecule. Dose-response evaluation across concentrations of 10, 40, 100, and 150 μg/mL yielded an IC_50_ of 35.09 ± 0.03 μg/mL after 24 h of treatment (Figure 10). Viability assays revealed a clear dose-dependent trend, with cell viability decreasing from approximately 75% at 10 μg/mL to 24.6% at 150 μg/mL concentration. The dose-response curve exhibited a hill slope of 2.575, indicative of a highly sensitive cellular reaction to varying concentrations, and a strong correlation coefficient (*R*^2^ = 0.9693), which supports the robustness of the data. Notably, a preincubated sample (100 μg/mL of the complex in medium for 4 days) resulted in 27.8% cell viability, closely paralleling the viability observed with freshly prepared 100 μg/mL (28.5%) and 150 μg/mL (24.6%) concentrations (Figure 11). This similarity provides powerful evidence that the cytotoxicity is attributable to the breakdown products in both fresh and predecomposed samples.  Figure 12 illustrates the morphological changes in cancer cells before and after 24 h treatment at 150 μg/mL. Under comparable conditions, cisplatin showed a substantially higher IC_50_ (> 200 μg/mL), indicating the superior cytotoxic potency of the complex's decomposition mixture within 24 h exposure [45]. Cytotoxicity likely arises from a mixture of vanadium species, dissociated ligands, or synergistic effects among multiple degradation products. Although the exact active species have yet to be fully identified, the robust and consistent cytotoxic activity suggests that the breakdown products may have stronger anticancer properties compared to the intact complex [44].

Vanadium complexes in biological environments exhibit significant structural modifications, including partially modified coordination complexes, hydrolyzed vanadium ions, and vanadium-coordinated biomolecules, such as modified or phosphate ions [46, 47]. Vanadium, in its various chemical forms, exhibits potent antitumor effects through diverse biological pathways, inducing DNA degradation, modulating cell division phases, activating cellular suicide mechanisms such as apoptosis and autophagy, preventing metastatic progression and invasive behavior, and compromising mitochondrial operations [48]. At higher doses, it disrupts normal cell function and causes mitochondrial dysfunction and apoptosis in healthy tissue. Therefore, the therapeutic window, the range in which cancer cells are affected while normal cells are not harmed, may be narrow and require precise dosing [49]. These features suggest a prodrug-like mechanism in which the complexes are converted to active cytotoxic species after biotransformation. This behavior is common among metal-based anticancer drugs.

### 3.5. Antimicrobial Activity

**Figure 13:**
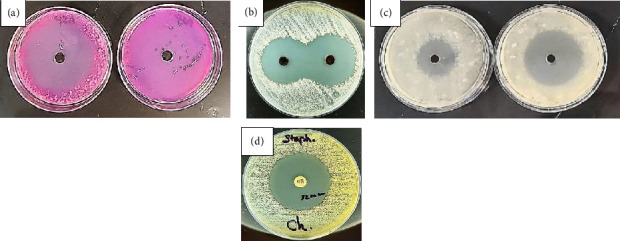
Antibacterial activities with 10 and 20 ppm solutions of [VO(L) (5-Cl-8-HQ)] against (a) *Asp. niger*, (b) *S. aureus*, (c) *E. faecalis*, and (d) chloramphenicol.

The antibacterial properties of the oxovanadium complex [VO(L) (5-Cl-8-HQ)] were tested at two concentrations (10 and 20 ppm) against *Staphylococcus aureus*, *Enterococcus faecalis*, and *Aspergillus niger* (Figure 13). The IZ diameters are listed in Table 2. The complex exhibited significant concentration-dependent antibacterial activity against all three organisms. The IZ for *S. aureus* increased from 32 mm to 36 mm, for *E. faecalis* from 24 mm to 31 mm, and from 41 mm to > 60 mm for *A. niger*. At both concentrations, the complex exhibited the highest efficacy against *A. niger* with the largest IZs (41 mm at 10 ppm and > 60 mm at 20 ppm). Compared to similar oxovanadium complexes reported in the literature [50, 51], the [VO(L) (5-Cl-8-HQ)] demonstrated notably stronger antibacterial activity with larger IZs. The unique structure of the complex, combining an oxovanadium center with a Schiff base ligand (sal-arginine) and chloroquinoline (5-Cl-8-HQ), likely contributes to its antimicrobial properties. Thus, each component may play a specific role.

- Oxovanadium center: Potential source of reactive oxygen species (ROS) [54].
- Schiff base ligand: Promoted cell permeation and metal chelation [55].
- Chloroquinoline: Lipophilicity for membrane penetration and proven antifungal activity [56]. These findings indicate that [VO(L) (5-Cl-8-HQ)] is a promising candidate for future studies on antimicrobial applications.

## 4. Conclusion

The multifunctional potential of the new oxovanadium(V) complex, [VO(L) (5-Cl-8-HQ)], derived from the Schiff base of L-arginine and salicylaldehyde and 5-chloroquinolin-8-ol, were investigated in this study. The complex was characterized using elemental analysis, PXRD, FTIR, UV-Vis, and ^1^H NMR spectroscopy, and cyclic voltammetry. As a pyridoxine sensor, electrochemical investigations demonstrated a quasireversible behavior with promising performance, exhibiting high sensitivity with an electrochemical rate constant of *k*_*e*_ = 0.133 s^−1^ in the linear range of detection of 1.0 × 10^−8^ to 1.0 × 10^−4^ mol·L^−1^, and a broad linear detection range of 4.24 × 10^−8^ mol·L^−1^. Biological evaluation revealed strong antimicrobial efficacy against gram-positive bacteria and the fungal strain *Aspergillus nige*r, as well as significant anticancer activity against the MDA-MB-231 breast cancer cell line, with an IC_50_ of 35.09 ± 0.03 μg/mL, and dose-dependent cytotoxicity. Evaluations of the stability of the complex under physiological conditions revealed a relatively fast breakdown, indicating that the decomposition products may play a role in determining some of the complex's cytotoxic and antimicrobial activities. This multipurpose vanadium complex provides a flexible platform for additional research in medicinal chemistry and biosensing applications by combining analytical sensing with therapeutic potential.

## Figures and Tables

**Table 1:** Melting point, elemental analysis, and spectroscopic data of the complex [VO(L) (5-Cl-8-HQ)].

Analysis	[VO(L) (5-Cl-HQ)]	

Melting point	193°C	

Elemental analysis found/(calculate)	C%	49.97/(50.54)
	N%	13.07/(13.39)
	H%	4.20/(4.24)

UV-Vis *λ* max (nm)	*π* ⟶ *π*^∗^	276
	*n *⟶* π*^∗^	305
	*Pπ *⟶* *3d	387

IR (*υ*/cm^−1^)	N-H	3342, 3198
	C-H	2941
	C=N	1667
	HC=N	1621
	C=O	1546
	C=C_ring_	1494, 1462
	C-O	1367
	C-N_Ar_	1309
	V=O	906
	V-O	625
	V-N	546

1H NMR *δ* (ppm)	H-Ar	6.78–7.48
	2H(CH_2_-CH_2_)	1–3.16
	1H(C=NH) _arginine_	8.77
	1H(N-H)	3.22
	2H(CH_2_-N-V)	4.42

**Table 2:** Antimicrobial activities of [VO(L) (5-Cl-8-HQ)] at 10 and 20 ppm of IZ.

[VO(L) (5-Cl-8-HQ)]	Microorganisms		
	*S. aureus*	*E. faecalis*	*Asp. niger*
Concentration at 10 ppm inhibition zone (mm)	32	24	41
Concentration at 20 ppm inhibition zone (mm)	36	31	> 60
Chloramphenicol inhibition zone (mm)	32	7.25 [52]	
Ketoconazole inhibition zone (mm)			11.9 [53]

Abbreviation: IZ, inhibition zone.

## Data Availability

The data that support the findings of this study are available on request from the corresponding author. The data are not publicly available due to privacy or ethical restrictions.
